# PD-1/PD-L1 inhibitor-induced hyponatremia: a real-world pharmacovigilance analysis using FAERS database

**DOI:** 10.3389/fimmu.2025.1561942

**Published:** 2025-06-16

**Authors:** Chao Tao, Bingyao Liu, Yan Dai, Jinyi Lv, Huanhuan He, Qian Ding, Kun Chen, Ke Wang, Liuxuan Yang, Xiaoqun Ren, Meiling Zhou

**Affiliations:** ^1^ Department of Pharmacy, The Affiliated Hospital, Southwest Medical University, Luzhou, China; ^2^ Department of Clinical Pharmacy, School of Pharmacy, Southwest Medical University, Luzhou, China; ^3^ Department of Radiology, West China Hospital Sichuan University Jintang Hospital, Chengdu, China; ^4^ Department of Rehabilitation, The Affiliated Hospital of Southwest Medical University, Luzhou, China; ^5^ Department of Clinical Pharmacy, The Third Hospital of Mianyang, Sichuan Mental Health Center, Mianyang, China

**Keywords:** immune checkpoint inhibitors, PD-1/PD-L1 inhibitors, hyponatremia, disproportionality analysis, pharmacovigilance, FAERS

## Abstract

**Background:**

With the increasing use of programmed cell death protein 1 and programmed cell death ligand 1 (PD-1/PD-L1) inhibitors in cancer treatment, hyponatremia has emerged as a notable adverse event associated with this class of drugs.

**Methods:**

We extracted adverse event reports related to PD-1/PD-L1 inhibitor-induced hyponatremia from the FDA Adverse Event Reporting System (FAERS) database, spanning from Q1–2004 to Q2 2024. The reports were analyzed for disproportionality using four methods: reporting odds ratio, proportional reporting ratio, Bayesian confidence propagation neural network, and multi-item gamma Poisson shrinker. Signals of hyponatremia associated with nivolumab, pembrolizumab, and atezolizumab were assessed at both the Standardized MedDRA Query and preferred term levels.

**Results:**

A total of 1,339 reports of hyponatremia involving 1,274 patients were identified, with nivolumab, pembrolizumab, or atezolizumab as the primary suspected drugs. All four methods consistently indicated positive signals for hyponatremia with these drugs. Hyponatremia induced by PD-1/PD-L1 inhibitors predominantly occurred in patients aged 45 and older, with a higher incidence in males. The median onset times were 42 days for nivolumab, 35 days for pembrolizumab, and 20 days for atezolizumab. Except for atezolizumab, the median onset times for hyponatremia induced by nivolumab and pembrolizumab differed across genders and age groups.

**Conclusion:**

This pharmacovigilance analysis reveals the association between PD-1/PD-L1 inhibitors and hyponatremia, offering valuable insights to refine treatment strategies and improve risk management for this AE.

## 1 Introduction

Cancer remains a major global public health challenge and continues to garner attention due to its rising incidence and mortality rates. Epidemiological studies suggest that nearly 20 million new cancer cases were diagnosed worldwide in 2022, with approximately 10 million cancer-related deaths. Projections indicate that by 2050, the annual global cancer cases will reach nearly 35 million, representing a 177% increase compared to 2022 (1). By 2070, the incidence is expected to double relative to the 2020 level (2). Programmed cell death protein 1 and programmed cell death ligand 1 (PD-1/PD-L1) inhibitors, as an important class of immune checkpoint inhibitors (ICIs), have become increasingly prominent in cancer treatment. These inhibitors function by blocking the interaction between PD-1 and PD-L1, thereby activating cytotoxic T lymphocytes, relieving immune suppression, enhancing the host immune response against tumor cells, and effectively inhibiting tumor growth and metastasis (3, 4). Despite their efficacy, PD-1/PD-L1 inhibitors are associated with a spectrum of adverse events (AEs), which may affect multiple organ systems. These AEs are closely linked to disease progression and survival outcomes, underscoring the importance of vigilant monitoring and management (5–8).

Hyponatremia, the most common electrolyte disturbance in clinical practice, serves as a critical prognostic indicator in cancer patients, closely linked to increased hospitalization rates and prolonged hospital stays (9). Drug-induced hyponatremia is defined as a serum sodium concentration below 135 mEq/L, with clinical manifestations that vary widely in severity depending on the rate of onset, duration, and degree of sodium depletion. Symptoms progress from fatigue, headache, nausea, vomiting, and muscle cramps to severe neurological complications such as seizures, confusion, and altered consciousness, with extreme cases potentially leading to coma or death. Studies have shown that hyponatremia is commonly observed among cancer patients receiving ICIs, especially PD-1/PD-L1 inhibitors (10–14). The development of PD-1/PD-L1 inhibitor-induced hyponatremia may involve multiple mechanisms, including the syndrome of inappropriate antidiuretic hormone secretion (SIADH) (15), immune-related endocrine disorders, and factors associated with significant fluid loss resulting from immune-related adverse events (irAEs) (10, 16). These mechanisms may act synergistically to contribute to the onset of hyponatremia. Given its prevalence and clinical significance, close monitoring for hyponatremia is important in cancer patients undergoing PD-1/PD-L1 inhibitor therapy.

The FDA Adverse Event Reporting System (FAERS) is a globally leading spontaneous reporting database that plays a pivotal role in monitoring the safety of marketed drugs and biologics. By leveraging advanced data mining algorithms, potential associations between drugs and AEs can be quantitatively assessed, enabling pharmacoepidemiological research and pharmacovigilance analysis. Although cases of hyponatremia have been reported in patients receiving PD-1/PD-L1 inhibitors in clinical practice (17–19), evidence from clinical trials and case reports remains limited due to small sample sizes (20). To date, no study has systematically investigated PD-1/PD-L1 inhibitor-induced hyponatremia using the FAERS database. To address this gap, this study performed a comprehensive pharmacovigilance analysis to evaluate the association between PD-1/PD-L1 inhibitors and hyponatremia using real-world data from FAERS. Data from Q1–2004 to Q2–2024 were screened, focusing on AE reports of hyponatremia where nivolumab, pembrolizumab, or atezolizumab were identified as the primary suspected drugs. Signals of hyponatremia related to these agents were quantitatively assessed using data mining algorithms, evaluating their distribution and strength of association at both the Standardized MedDRA Query (SMQ) and preferred term (PT) levels. Additionally, we analyzed the median onset time of drug-induced hyponatremia. This study provides valuable insights into the prevention and management of PD-1/PD-L1 inhibitor-induced hyponatremia, supporting the development of precise treatment strategies and promoting safer, more rational drug use.

## 2 Methods

### 2.1 Data source

This study utilized data from the FAERS database to perform a pharmacovigilance analysis of hyponatremia associated with PD-1/PD-L1 inhibitors. The FAERS database is a publicly accessible spontaneous reporting system that compiles safety reports on marketed drugs and biologics from countries worldwide. These reports are submitted by healthcare professionals, drug manufacturers, patients, and other stakeholders such as attorneys. Since 2004, the FAERS database has been publicly available and updated quarterly. It includes seven datasets: demographic and management information (DEMO), drug information (DRUG), adverse event information (REAC), patient outcome information (OUTC), report source information (RPSR), drug therapy start and end dates (THER), and indication/diagnosis information (INDI). For this study, we extracted data from FAERS covering the period from Q1–2004 to Q2 2024, following FDA guidelines and official recommendations. Data selection and processing were performed using SAS 9.4 software.

### 2.2 Identification of relevant reports

In this study, AEs were coded using PTs from the Medical Dictionary for Regulatory Activities (MedDRA, version 26.1), which provides standardized and precise descriptions of medical conditions (21). Each PT is linked to multiple high-level terms, high-level group terms, and system organ classes (22). Additionally, SMQ is a built-in tool within MedDRA that consists of collections of PTs representing similar medical conditions, facilitating the identification of relevant safety reports. To ensure the specificity and accuracy of AE reports, we referenced the “Hyponatremia (SMQ)” entry in MedDRA version 26.1. A narrow-scope SMQ search was conducted to identify relevant PTs for hyponatremia and extract AE reports listing PD-1/PD-L1 inhibitors as the primary suspected drugs. According to this SMQ definition, hyponatremia is defined as a serum sodium concentration below 135 mEq/L. The specific PTs included in this analysis are detailed in **Table 1**.

**Table 1:** PTs contained in the narrow-scope search of “Hyponatremia (SMQ)”.

MedDRA code	Preferred terms
10021036	Hyponatremia
10005802	Blood sodium decreased
10053198	Inappropriate antidiuretic hormone secretion
10005800	Blood sodium abnormal
10069350	Osmotic demyelination syndrome
10021037	Hyponatremic syndrome
10070604	Rapid correction of hyponatremia
10066151	Hyponatremic encephalopathy
10074867	Hypoosmolar state
10049222	Neonatal hyponatremia
10005335	Blood antidiuretic hormone increased
10002776	Antidiuretic hormone abnormality
10075865	Hyponatremic coma
10005332	Blood antidiuretic hormone abnormal
10014149	Ectopic antidiuretic hormone secretion

### 2.3 Analytical methods

This study analyzed the clinical characteristics of patients experiencing hyponatremia induced by PD-1/PD-L1 inhibitors, encompassing report year, patient demographics (age and sex), reporter type, country of origin, and patient outcomes. A disproportionality analysis was performed using data mining algorithms to quantitatively detect AE signals in large pharmacovigilance databases, identifying potential associations with PD-1/PD-L1 inhibitors (23, 24). To compare the frequency of these AEs with their background frequency, a classic 2 × 2 contingency table (**Supplementary Table S1**) was utilized to establish statistical associations.

**Figure 1:**
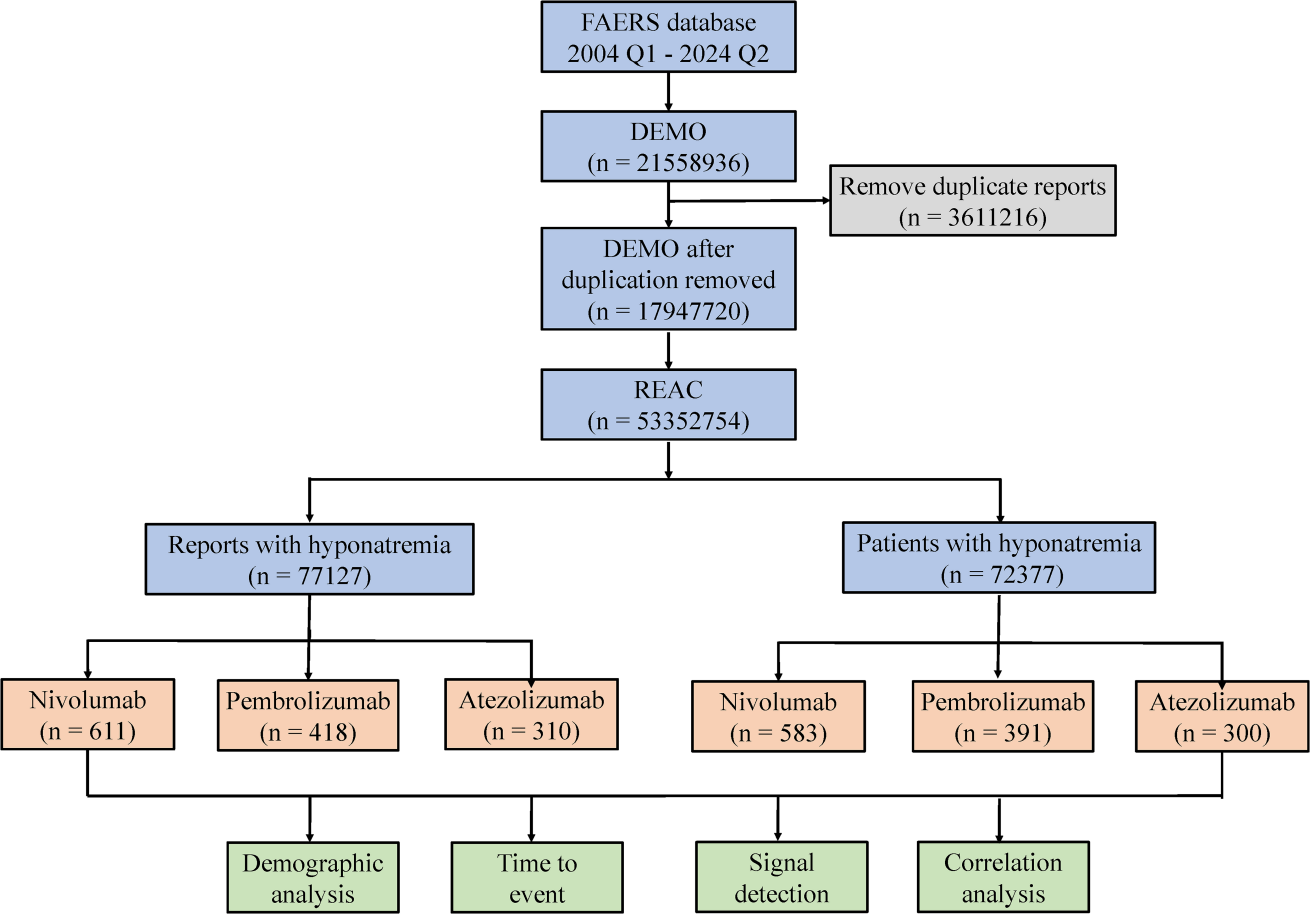
The flow chart of the study.

Four methods were employed to evaluate signal strength and potential AE risk: reporting odds ratio (ROR), proportional reporting ratio (PRR), Bayesian confidence propagation neural network (BCPNN), and multi-item gamma Poisson shrinker (MGPS) (25, 26). The formulas and evaluation criteria are outlined in **Table 2**. The ROR, PRR, information component (IC), and empirical Bayes geometric mean (EBGM) values were used as quantitative metrics to compare AE risks across different drugs. Higher values reflect stronger correlations and an elevated risk of hyponatremia linked to the specific drug. Furthermore, the onset time of drug-induced hyponatremia was determined by calculating the interval between the start date of the primary suspected medication and the date of the first reported AE. This study focused on three widely used and frequently reported PD-1/PD-L1 inhibitors: nivolumab, pembrolizumab, and atezolizumab. A process diagram illustrating the study workflow is presented in **Figure 1**.

**Table 2:** Overview of algorithms utilized for signal detection.

Algorithms	Formulas	Criteria
ROR	$\text{ROR = }\frac{\text{ad}}{\text{bc}}$	Lower limit of 95% CI > 1, a ≥ 3
	${95\% CI = e}^{In\left(ROR\right)\pm 1.96{\left(\frac{1}{a}+\frac{1}{b}+\frac{1}{c}+\frac{1}{d}\right)}^{0.5}}$	
PRR	$\text{PRR = }\frac{\text{a/(a+b)}}{\text{c/(c+d)}}$	PRR ≥ 2, χ^2^ ≥ 4, a ≥ 3
	$\chi^{2}\text{= }\frac{{\left(\text{ad-bc}\right)}^{2}\text{(a+b+c+d)}}{\text{(a+b)(c+d)(a+c)(b+d)}}$	
BCPNN	${\text{IC = log}}_{2}\frac{\text{a}\left(\text{a+b+c+d}\right)}{\left(\text{a+c}\right)\left(\text{a+b}\right)}$	IC025 > 0
	${\text{IC025 = E(IC)-2[V(IC)]}}^{0.5}$	
MGPS	$\text{EBGM = }\frac{\text{a(a+b+c+d)}}{\text{(a+c)(a+b)}}$	EBGM05> 2, a > 0
	$95\text{\% C= }{\text{e}}^{\text{ln}\left(\text{EBGM}\right)\;\pm \;1.96\;{\left(\frac{1}{\text{a}}\;+\;\frac{1}{\text{b}}\;+\;\frac{1}{\text{c}}\;+\;\frac{1}{\text{d}}\right)}^{0.5}}$	

CI, confidence interval; χ^2^, chi-squared; IC, information component; E(IC), the IC expectations; V(IC), the variance of IC; EBGM, empirical Bayes geometric mean.

## 3 Results

### 3.1 Descriptive analysis

**Figure 2:**
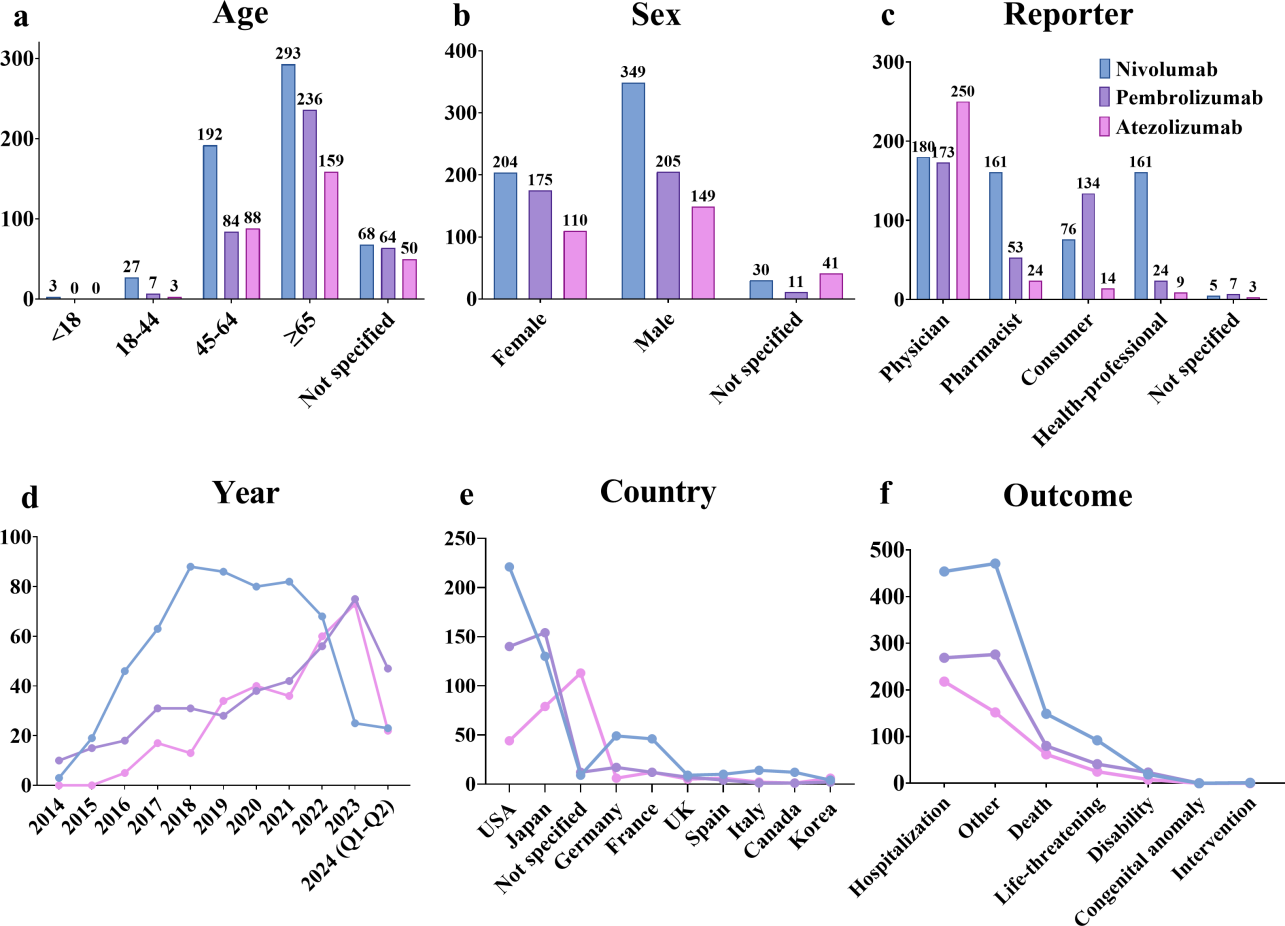
AE reports related to hyponatremia induced by PD-1/PD-L1 inhibitors in the FAERS database. **(a)** Age distribution of patients. **(b)** Gender distribution of patients. **(c)** Distribution of reporters by occupation. **(d)** Annual trends in AE reports. **(e)** Top 10 countries by number of reports. **(f)** Distribution of patient outcomes.

The results revealed that from Q1–2004 to Q2 2024, a narrow-scope search of “Hyponatremia (SMQ)” in the FAERS database identified 1,339 AE reports listing PD-1/PD-L1 inhibitors as the primary suspected drugs, involving 1,274 patients. When categorized by age, patients over 65 years accounted for the highest proportion (54.00%) of hyponatremia cases (**Figure 2a**). In terms of gender distribution, male patients exhibited a significantly higher rate of hyponatremia (55.18%) compared to females (38.38%) (**Figure 2b**). Physicians constituted the largest group of reporters (47.33%), followed by pharmacists (18.68%) (**Figure 2c**). Reporting trends varied among drugs: for nivolumab, reports of hyponatremia increased annually until 2018, followed by a gradual decline from 2018 to 2022, and a sharp decrease thereafter. In contrast, reports related to pembrolizumab and atezolizumab demonstrated a general upward trend after market approval (**Figure 2d**). Geographically, most reports originated from the USA (31.79%) and Japan (28.49%). Specifically, reports on nivolumab were more frequent in the USA, whereas pembrolizumab and atezolizumab were more prevalent in Japan (**Figure 2e**). Regarding patient outcomes, initial or prolonged hospitalization was the most frequently reported, accounting for 40.20% of all cases (**Figure 2f**).

### 3.2 Distribution of AE onset time

**Figure 3:**
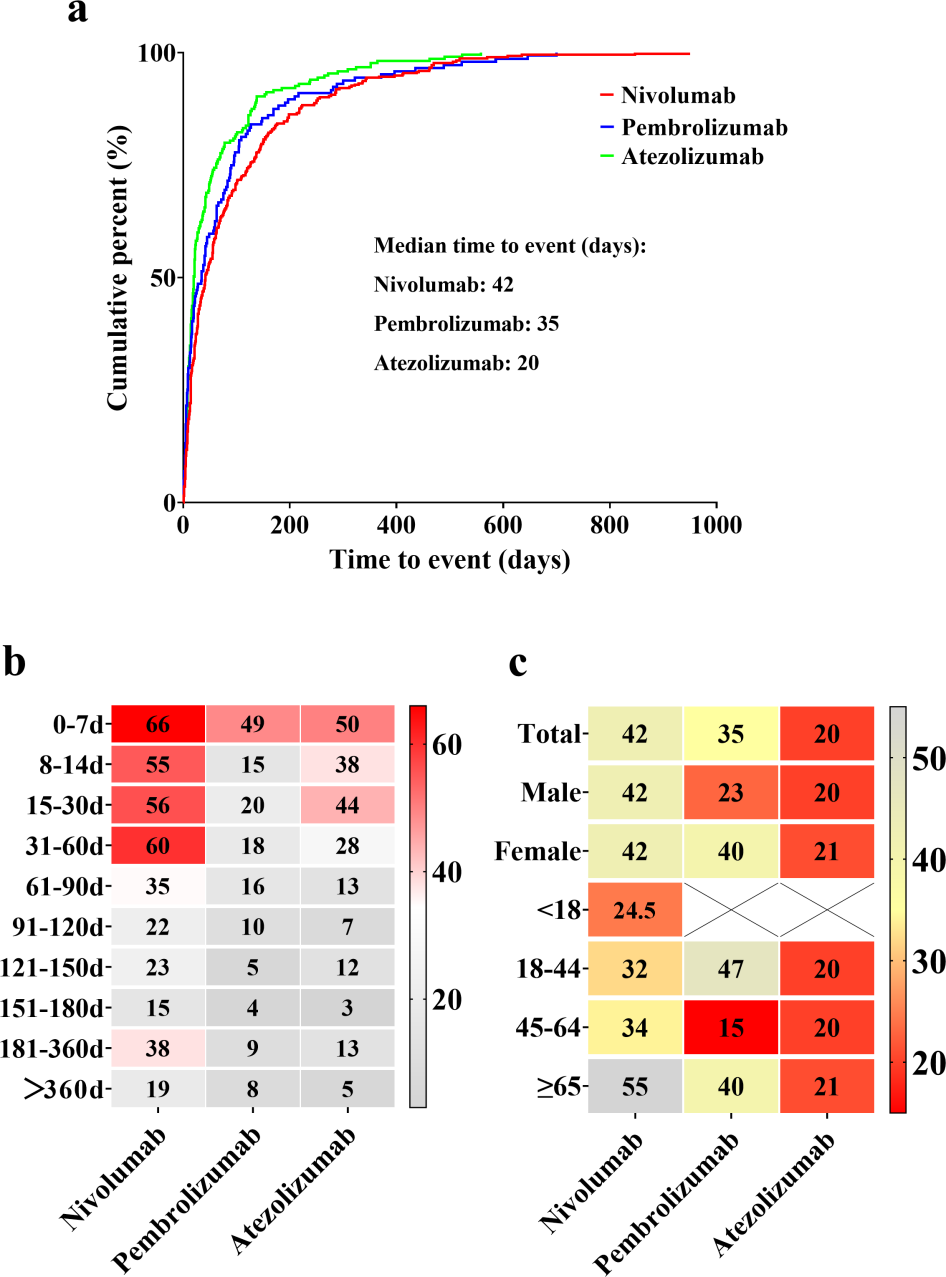
Overview of median onset times for hyponatremia associated with nivolumab, pembrolizumab, and atezolizumab. **(a)** Median onset times of drug-induced hyponatremia. **(b)** Proportional distribution of hyponatremia incidence across different time intervals. Red blocks indicate significant associations between hyponatremia occurrence and specific time intervals, while gray blocks represent weaker associations. **(c)** Median onset times for hyponatremia incidence among different genders and age groups. Red blocks highlight shorter median times, whereas gray blocks correspond to longer median times.

The median onset times for hyponatremia are shown in **Figure 3a**: 42 days for nivolumab, 35 days for pembrolizumab, and 20 days for atezolizumab. The peak onset periods also varied, with nivolumab peaking within 0–60 days, pembrolizumab within 0–7 days, and atezolizumab within 0–30 days (**Figure 3b**). For nivolumab, the median onset time was identical for both male and female patients (42 days), with a trend toward longer onset times in older age groups. For pembrolizumab, the median onset time was 23 days in male patients and 40 days in females, indicating a difference of 17 days. For atezolizumab, the median onset times were similar between sexes, with a difference of only 1 day. Notably, the shortest median onset (15 days) was observed in patients aged 45–64 years treated with pembrolizumab. For atezolizumab, the median onset time remained relatively consistent across all age groups (**Figure 3c**).

### 3.3 Proportional distribution of drugs in AE reports

**Figure 4:**
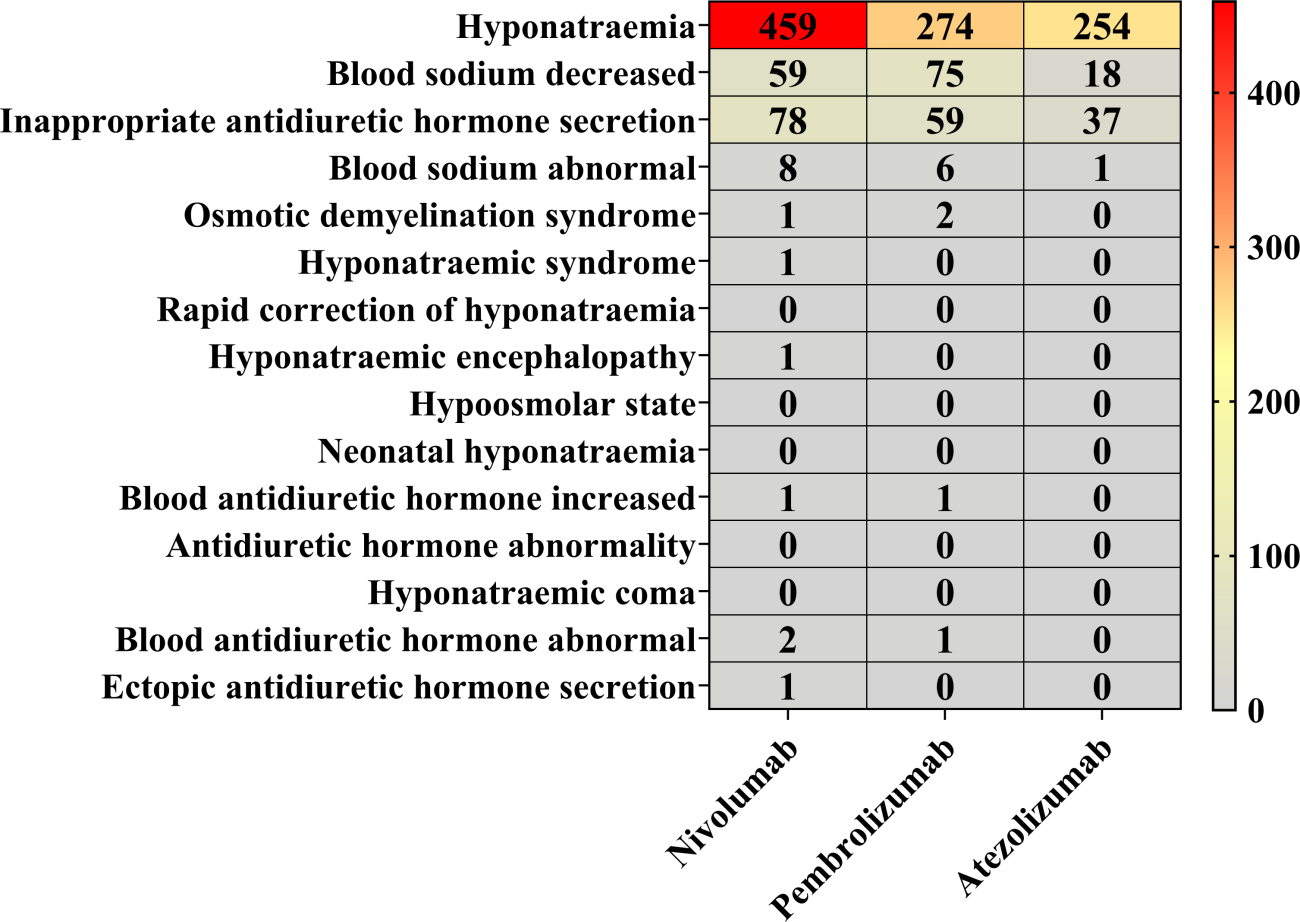
Proportional reporting of AEs at the SMQ and PT levels. Red blocks demonstrate significant associations between hyponatremia and the respective PTs, whereas gray blocks indicate non-significant associations.

The proportional distribution of AE reports for nivolumab, pembrolizumab, and atezolizumab was analyzed at both the SMQ and PT levels (**Figure 4**). Nivolumab was associated with reports in 10 PT categories, pembrolizumab in 7 PT categories, and atezolizumab in 4 PT categories. Among these, “hyponatremia” (PT) accounted for the highest number of reports across all three drugs, with 459 reports for nivolumab (75.12% of nivolumab-related reports), 274 reports for pembrolizumab (65.55% of pembrolizumab-related reports), and 254 reports for atezolizumab (81.94% of atezolizumab-related reports). Other commonly reported PTs included “blood sodium decreased”, “inappropriate antidiuretic hormone secretion”, and “blood sodium abnormal”. In contrast, the remaining PTs were associated with three or fewer reports for each drug.

### 3.4 AE signal detection results

**Figure 5:**
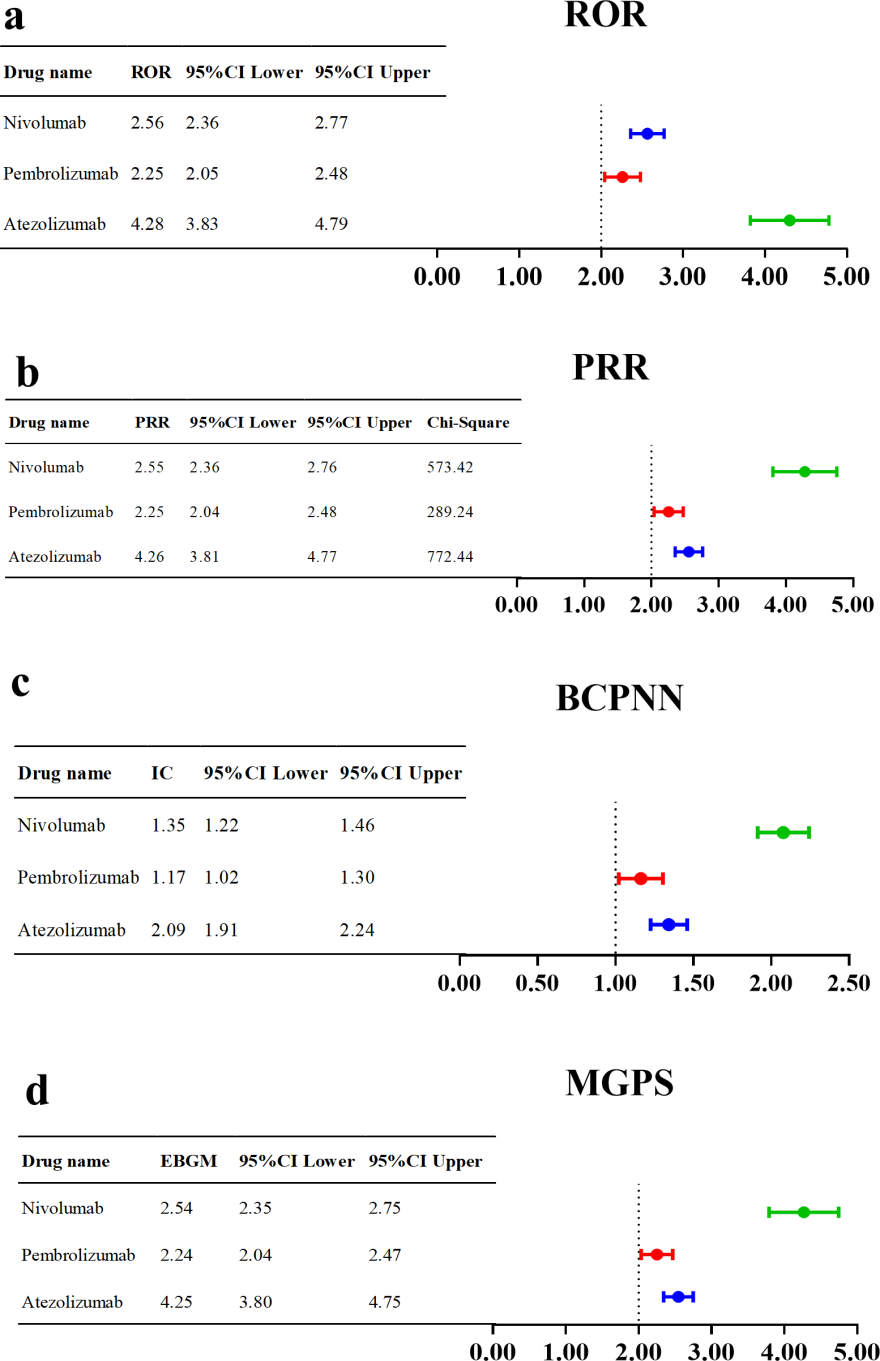
Signal detection analysis of hyponatremia associated with PD-1/PD-L1 inhibitors using **(a)** ROR, **(b)** PRR, **(c)** BCPNN, and **(d)** MGPS methods.

To assess the potential risk of hyponatremia induced by nivolumab, pembrolizumab, and atezolizumab, a comprehensive AE signal detection analysis was conducted, with the results presented in **Figure 5**. All three drugs showed positive signals for hyponatremia: nivolumab (n = 611, ROR = 2.56, PRR = 2.55, χ² = 573.42, IC = 1.35, IC025 = 1.22, EBGM = 2.54), pembrolizumab (n = 418, ROR = 2.25, PRR = 2.25, χ² = 289.24, IC = 1.17, IC025 = 1.02, EBGM = 2.24), atezolizumab (n = 310, ROR = 4.28, PRR = 4.26, χ² = 772.44, IC = 2.09, IC025 = 1.91, EBGM = 4.25). All four detection methods (ROR, PRR, BCPNN, and MGPS) consistently identified positive signals for hyponatremia associated with these PD-1/PD-L1 inhibitors (**Supplementary Figure S1**). Additionally, a review of the drug instructions for these drugs revealed that only the instructions for pembrolizumab did not list hyponatremia as an AE or mention it in the precautions.

## 4 Discussion

With growing public awareness of adverse reactions associated with drug therapies, drug-induced hyponatremia has become a noteworthy safety concern for both patients and healthcare providers. To address the limitations of clinical trials and case reports in investigating PD-1/PD-L1-induced hyponatremia, our study is the first to leverage the FAERS database and apply multiple disproportionality analysis methods, including ROR, PRR, BCPNN, and MGPS, to quantify the association between PD-1/PD-L1 inhibitors and hyponatremia. Higher signal detection values demonstrated a stronger association, indicating an increased risk of hyponatremia in cancer patients treated with these drugs. We systematically analyzed 1,339 hyponatremia-related reports involving PD-1/PD-L1 inhibitors (nivolumab, pembrolizumab, and atezolizumab) as the primary suspected drugs. The basic characteristics of the patients and reports were described, and differences in the onset time of hyponatremia associated with these inhibitors were explored. Additionally, the distribution of PD-1/PD-L1 inhibitors was examined at both the SMQ and PT levels to provide a more detailed safety profile.

This study found that hyponatremia induced by PD-1/PD-L1 inhibitors predominantly affects patients aged 45 and older. Notably, according to the 2022 global cancer statistics, 10.3 million new cancer cases were reported in males (51.50%) and 9.7 million in females (48.50%), with no significant difference in incidence between the sexes (1). Similarly, previous studies have suggested that overall cancer incidence is approximately equal between males and females when not stratified by cancer type (27). However, our analysis demonstrated a higher frequency of AE reports in male patients, with a gender difference of 16.80%. Drug-specific analyses further supported this trend, as nivolumab, pembrolizumab, and atezolizumab each showed more reports in male patients. These findings underscore the importance of greater vigilance regarding hyponatremia in male patients undergoing PD-1/PD-L1 inhibitor therapy. From 2015 to 2022, reports associated with nivolumab surpassed those for pembrolizumab and atezolizumab. However, in 2023, reports for nivolumab declined sharply. This trend may be attributed to market competition and evolving treatment strategies. The emergence of alternative therapies, such as atezolizumab or newer ICIs, likely contributed to the reduced use of nivolumab. Additionally, the accumulation of safety data may have influenced prescribing behaviors. Early reports of specific AEs associated with nivolumab might have led physicians to prefer alternative options perceived as safer, thereby mitigating the risk of adverse outcomes. Furthermore, our data indicate that the clinical outcomes of AE reports for nivolumab, pembrolizumab, and atezolizumab were severe, with proportions of 95.20%, 93.61%, and 97.67%, respectively. These findings highlight the significant impact of hyponatremia linked to PD-1/PD-L1 inhibitors in cancer patients, emphasizing the need for vigilant monitoring and risk mitigation in clinical practice.

This study systematically summarized the onset time of hyponatremia induced by nivolumab, pembrolizumab, and atezolizumab. Overall, nivolumab-induced hyponatremia predominantly occurs within the first two months of treatment, as reported in multiple case reports and observational studies (16, 18, 28–30). Additionally, some studies have reported cases occurring within four months after nivolumab initiation (13, 31, 32). There is also a documented case of severe hyponatremia developing within 24 h after the first dose (17). The onset pattern of atezolizumab-induced hyponatremia is consistent with existing literature, typically occurring within the first 1.5 months of treatment (33). Case reports on pembrolizumab suggest that hyponatremia may emerge three to four months after treatment initiation (34–37). However, in our study, pembrolizumab-induced hyponatremia showed no clear clustering in time to onset, with a median onset time of 35 days. This discrepancy may be attributed to the inherent variability and limited sample sizes in case report studies. In our study, no significant differences in the median onset time of hyponatremia were observed between genders for nivolumab and atezolizumab. However, a notable gender difference was found for pembrolizumab-induced hyponatremia, with males experiencing onset earlier than females. These findings suggest that healthcare providers should consider gender differences, especially in monitoring male patients, when evaluating hyponatremia risk in those treated with pembrolizumab. Regarding age-related trends, the median onset time for nivolumab-induced hyponatremia increases with age, indicating a need for heightened vigilance in middle-aged and older patients receiving nivolumab. For pembrolizumab, the median onset time is shortest in patients aged 45–64 years, with a median of just 15 days. This highlights the necessity for close monitoring of hyponatremia risk in this age group, especially within the first two weeks of treatment initiation. Compared with nivolumab and pembrolizumab, atezolizumab is associated with a shorter median onset time for hyponatremia across all age groups and genders, underscoring the need for increased vigilance during its use.

Although this study primarily focused on AE reports of hyponatremia in which nivolumab, pembrolizumab, or atezolizumab were identified as the primary suspected drugs, a total of 71 concomitant medications associated with PD-1/PD-L1 inhibitors were also analyzed using the shrinkage measure method for signal detection (38, 39). Detailed results are presented in **Supplementary Table S2**. Only a few drug combinations had more than 10 reports, including: nivolumab and ipilimumab (n = 107, Ω = –0.89, Ω025 = –1.16); nivolumab and cabozantinib (n = 12, Ω = –0.51, Ω025 = –1.33); pembrolizumab and lenvatinib (n = 81, Ω = –0.23, Ω025 = –0.54); and atezolizumab and bevacizumab (n = 17, Ω = –1.68, Ω025 = –2.36). All of these combinations yielded Ω025 values below zero, indicating negative signals. Among the 71 concomitant medications analyzed, only two showed positive signals. Previous studies have suggested that small sample sizes may impair the performance of signal detection algorithms, making it difficult to distinguish true safety signals from background noise (40). Therefore, due to the limited number of reports and corresponding statistical limitations, we currently lack sufficient evidence to explore potential associations between PD-1/PD-L1 inhibitors and concomitant medications in the development of hyponatremia.

Existing studies have established an association between nivolumab and hyponatremia in the treatment of various cancers, including epithelial cancer (41), squamous cell carcinoma (42–44), cervical cancer (45), squamous non-small cell lung cancer (32), cholangiocarcinoma (46), melanoma (16), and rectal adenocarcinoma (31). Similarly, pembrolizumab has been implicated in causing hyponatremia during the treatment of non-small cell lung cancer (47, 48), epithelial cancer (49, 50), and non-muscle invasive bladder cancer (51). In comparison, evidence linking atezolizumab to hyponatremia is relatively limited, with reports primarily focused on non-small cell lung cancer and hepatocellular carcinoma (33). The mechanisms underlying PD-1/PD-L1 inhibitor-induced hyponatremia remain not fully understood, but it is widely believed that multiple mechanisms are involved (10, 15, 16). A commonly recognized mechanism is the SIADH, which is considered a major cause of hyponatremia induced by these inhibitors (15). Additionally, endocrine disorders associated with irAEs, such as pituitary insufficiency, adrenal insufficiency, and hypothyroidism, play an important role in the pathogenesis of hyponatremia (13, 14, 32–34, 52). Immune-mediated conditions, including hypophysitis, isolated adrenocorticotropic hormone deficiency, renal injury, and thyroiditis, are frequently reported during PD-1/PD-L1 inhibitor treatment (12, 53, 54). Moreover, immune-mediated enteritis may cause substantial fluid loss and hypovolemia, potentially leading to hemodynamic instability and subsequent hypovolemic hyponatremia (10, 16). It is important to note that these mechanisms may interact synergistically, collectively contributing to the development of hyponatremia. For instance, irAE-induced adrenal insufficiency may contribute to both SIADH and hypovolemia, thereby exacerbating electrolyte imbalances. Furthermore, individual patient characteristics, such as age, baseline renal function, and preexisting endocrine disorders, may influence the susceptibility to PD-1/PD-L1 inhibitor-induced hyponatremia. Those with adrenal insufficiency or thyroid dysfunction may be at an increased risk, necessitating closer monitoring. In patients receiving PD-1/PD-L1 inhibitors, the development of SIADH, endocrine disorders related to irAEs, or other conditions discussed above warrants heightened vigilance for the onset of hyponatremia.

Although this study provides a comprehensive analysis of PD-1/PD-L1 inhibitor-induced hyponatremia using a large pharmacovigilance database, it still has certain limitations. First, the reports in the FAERS database may be incomplete or duplicated. Additionally, these reports can be submitted by healthcare professionals, patients, lawyers, and other non-medical personnel, which introduces the possibility of data inaccuracies. Second, while large pharmacovigilance databases can reveal statistical associations between PD-1/PD-L1 inhibitors and hyponatremia, they do not establish direct causal relationships. Long-term prospective studies are required to validate these connections and elucidate causal mechanisms. Third, the FAERS database lacks information on pembrolizumab and atezolizumab use in patients under 18 years of age, leading to gaps in the dataset for this age group. Finally, cancer patients often receive combination therapies, particularly older patients with multiple comorbidities. The potential interactions between drugs and their collective impact on the risk of hyponatremia require further investigation to ensure the rational use of PD-1/PD-L1 inhibitors.

## 5 Conclusion

In conclusion, this study systematically analyzed FAERS data from Q1–2004 to Q2 2024, providing a comprehensive evaluation of hyponatremia induced by PD-1/PD-L1 inhibitors. A total of 1,339 hyponatremia-related reports, in which PD-1/PD-L1 inhibitors were the primary suspected drugs, involving 1,274 patients, were identified and summarized. Hyponatremia primarily occurs in patients aged 45 and older, with a significantly higher incidence in males. Differences in the median onset time of hyponatremia were evident for nivolumab and pembrolizumab in terms of gender and age, while atezolizumab exhibited no such variations. Despite certain limitations, our findings provide important real-world evidence on the risk of hyponatremia induced by PD-1/PD-L1 inhibitors. This study provides valuable insights for healthcare professionals in recognizing, preventing, and managing this AE. By contributing clinical evidence, this research aims to enhance the safety of drug therapies and support the optimization of clinical practices for cancer treatment.

## Data Availability

The original contributions presented in the study are included in the article/**Supplementary Material**. Further inquiries can be directed to the corresponding authors.
