# Supplementary material for: PD-1/PD-L1 inhibitor-induced hyponatremia: a real-world pharmacovigilance analysis using FAERS database
Source: Front Immunol. 2025 Jun 16;16:1561942. doi: 10.3389/fimmu.2025.1561942 (PMC12206762; doi:10.3389/fimmu.2025.1561942)
Supplement: Supplementary file 1 [file DataSheet1.docx]

**Supplementary material for**

**PD-1/PD-L1 Inhibitor-Induced Hyponatremia: A Real-World Pharmacovigilance Analysis Using FAERS Database**

**Table S1**. The classic two-by-two contingency table.

|  | Number of reports for target AE | Number of reports for other AEs | Total |
| --- | --- | --- | --- |
| Target drug | a | b | a + b |
| Other drugs | c | d | c + d |
| Total | a + c | b + d | a + b + c + d |


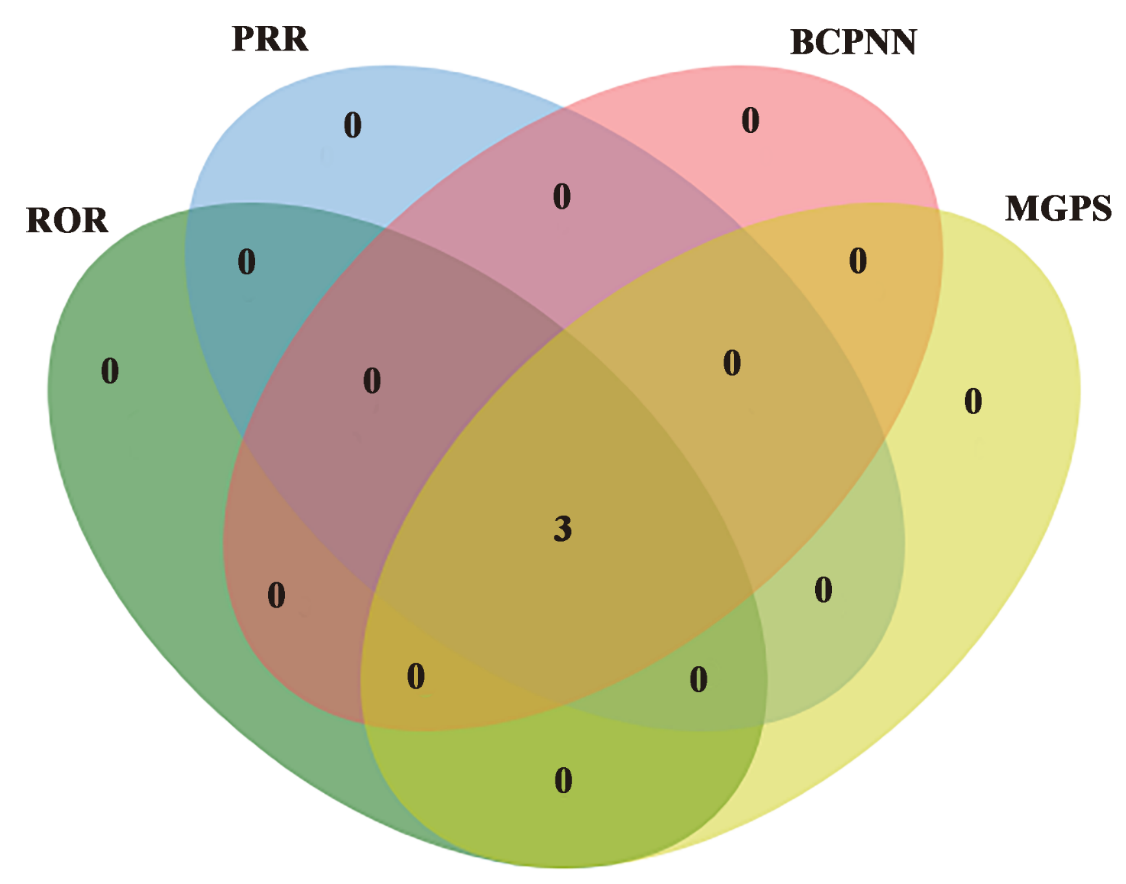


**Figure S1.** The number of positive drugs identified by ROR, PRR, BCPNN, and MGPS methods.
